# Ultrasensitive Detection of Pb^2+^ Based on a DNAzyme and Digital PCR

**DOI:** 10.1155/2019/3528345

**Published:** 2019-01-02

**Authors:** Tao Zhang, Cong Liu, Wuping Zhou, Keming Jiang, Chenyu Yin, Cong Liu, Zhiqiang Zhang, Haiwen Li

**Affiliations:** ^1^Key Lab of Bio-Medical Diagnostics, Suzhou Institute of Biomedical Engineering and Technology, Chinese Academy of Sciences, Suzhou 215163, China; ^2^School of Engineering Science, University of Science and Technology, Hefei, China

## Abstract

In this study, an ultrasensitive detection method for aqueous Pb^2+^ based on digital polymerase chain reaction (dPCR) technology and a Pb^2+^-dependent DNAzyme was developed. In the presence of Pb^2+^, the Gr-5 DNAzyme was activated and catalyzed the hydrolytic cleavage of the substrate strand, resulting in an increase in the amount of template DNA available for dPCR and a resultant change in the number of droplets showing a positive signal. Moreover, the detection system was found to be sensitive and stable in environmental sample detection. In summary, an ultrasensitive quantitative detection method for Pb^2+^ within environmental substrates was established.

## 1. Introduction

Lead ions (Pb^2+^), a major heavy metal pollutant, are a widespread and highly toxic contaminant in the environment. Pb^2+^ can accumulate in the human body, and exposure to very small amount of Pb^2+^ can lead to serious damage to the human brain and central nervous system, especially in children [1–3]. Various analytical methods have been developed for Pb^2+^ detection, such as atomic absorption spectrometry [4], atomic emission spectrometry, inductively coupled plasma atomic emission spectrometry [4, 5], inductively coupled plasma mass spectrometry [6], and X-ray fluorescence spectrometry [7]. In recent years, many new methods for Pb^2+^ detection have been developed, such as colorimetric [8], electrochemical [9, 10], fluorometric [11, 12], surface-enhanced Raman scattering [13, 14], and dynamic light scattering techniques [15]. One approach is based on the catalytic activity of DNAzymes [10, 14, 16–20]. The activity of DNAzymes, which are DNA-based catalysts, requires metal ions. Once activated by cofactors, the DNAzyme can induce chemical transformations and cleave DNA specifically. DNAzymes were first discovered via an in vitro selection process for RNA cleavage in the presence of Pb^2+^, and they have shown excellent selectivity for Pb^2+^ [21]. There are many advantages of DNAzymes as metal ion sensors. First, DNAzymes can be obtained with minimal information about metal ion-binding sites. Second, DNAzyme selection is more efficient than aptamer selection because of its higher separation efficiency. Finally, DNAzymes are easier to synthesize. Consequently, many DNAzyme-based sensors have been developed in the past few years. For example, lead-specific DNAzymes are known to cleave at adenosine ribonucleotide (rA) sites on partially complementary DNA substrates in the presence of Pb^2+^. Typical Pb^2+^-specific DNAzyme-based sensors utilize the enzymatic activity of the Gr-5 and “8–17” DNAzymes on a target element to produce a fluorescent or colorimetric output [10, 22, 23]. However, DNAzyme-based methods also have inherent drawbacks, such as high detection limits and high background signals. Recently, polymerase chain reaction (PCR) has been introduced combining with DNAzymes so that PCR methods could be used to improve the sensitivity and specificity of Pb^2+^ detection techniques.

Droplet digital PCR (ddPCR), a biotechnological refinement of conventional PCR methods, provides high-precision absolute quantification of nucleic acid target sequences and has wide-ranging applications for both research and clinical diagnostic uses [24–26]. First, the PCR mix is partitioned into thousands of water-in-oil droplets. Each droplet contains one or fewer copies of the target DNA. After end-point PCR amplification, each partition is checked for fluorescence with a binary readout of 0 (presence of PCR product) or 1 (absence of PCR product). Finally, absolute quantification of the target DNA molecules in the original sample can be directly calculated based on the Poisson distribution [24, 27, 28]. ddPCR has several benefits for nucleic acid quantification, including unparalleled precision, increased signal-to-noise ratio, removal of PCR efficiency bias, and simplified quantification, allowing a more reliable collection and more sensitive measurement of nucleic acid amounts. The method has been demonstrated to be useful for studying variations in gene sequences and is regarded as an improved nucleic acid detection technique compared with real-time PCR [29–32]. The absolute quantification of lead ions was essential for the risk assessment of lead ions. However, in methods such as colorimetric and electrochemical techniques, the quantitative detection of target ions could only be achieved through the analysis of standard curves which may be affected by the operation or the data analysis [33]. Previous studies have shown that when Pb^2+^ is present in the solution, it binds to the Gr-5 DNAzyme and facilitates the cleavage of the phosphodiester bond of the internal RNA base (rA) by the enzyme strand, and the amount of Pb^2+^ and cleavaged DNA is about one to one ratio. Thus, through absolute quantification of cleavaged DNA by droplet digital PCR, the absolute quantitative detection of Pb^2+^ can be achieved.

Herein, we report an ultrasensitive detection method for Pb^2+^ based on a DNAzyme biosensor and digital PCR (dPCR). A Pb^2+^-dependent Gr-5 DNAzyme was selected and immobilized on the inner wall of a plate [34]. The addition of aqueous Pb^2+^ to the plate leads to cleavage of the substrate DNA at the rA site by the Gr-5 DNAzyme, resulting in the release of the template DNA. Consequently, the PCR amplification signal varies according to the amount of template available for amplification, which in turn depends on the concentration of aqueous Pb^2+^. The method takes advantage of the selectivity of the DNAzyme and the sensitivity of dPCR. Moreover, this method can detect as low as 500 pM Pb^2+^ and can be used for analyzing complex assay mixtures such as environmental water samples.

## 2. Materials and Methods

### 2.1. Oligonucleotides and Reagents

Oligonucleotide sequences were purchased from Sangon Biotech Co., Ltd. (Shanghai, China), and the sequences are shown in Supplemental Table 1 as described previously [35]. Enzyme-labeled plates pretreated with streptavidin were purchased from Haili Biotech Co. Ltd. (Suzhou, China). The reagents related to ddPCR were purchased from Bio-Rad (Pleasanton, CA, USA). Lead ions and mercury ions were obtained from the National Standard Substances Center (Beijing, China). Zn(NO_3_)_2_, MgCl_2_, FeCl_2_, CuCl_2_, KCl, and CaCl_2_ were purchased from Sigma-Aldrich (St. Louis, MO, USA). Other chemicals were of analytical grade, and double-distilled water was used throughout the experiments.

### 2.2. DNAzyme Cleavage Assay

An enzyme-labeled plate pretreated with streptavidin was washed with PBST (10 mM PBS, pH 7.2, 0.05% Tween-20). Then, 50 *µ*L of a mixture of Gr-5 DNAzyme and substrate DNA at the appropriate concentration was added to PCR tubes. Gr-5 DNAzyme was fixed on the plate through biotin-streptavidin interaction and hybridized with the substrate DNA at 37°C for 30 min. Next, the plate was washed with hybridization buffer (750 mM NaCl, 75 mM, C_6_H_5_Na_3_O_7_, pH 8.0), and Pb^2+^ was added to the plate. After incubation at 37°C for 30 min, the plate was washed with hybridization buffer, and the cleaved DNA was collected for digital PCR (dPCR).

### 2.3. Digital PCR Assay

Digital PCR assay was operated according to the instruction of Bio-Rad. Each reaction mixture contained 10 *μ*L of ddPCR master mix for probe (Bio-Rad), 0.5 *μ*M reverse and forward primers, 1 *μ*L of template, and 7 *μ*L of distilled water. The cleaved DNA from the DNAzyme cleavage assay was used as the template DNA and was serially diluted to ensure the proper copy number range. Then, the mixture was loaded onto a droplet generation cartridge for droplet generation. Droplets were then collected and amplified. The PCR amplification conditions were as follows: 95°C for 5 min, 40 cycles of denaturation for 10 s at 95°C, and annealing for 60 s at 60°C and finally 10 min at 98°C. The amplified droplets were detected by a dPCR system, and the data were analyzed using Right PCR software.

### 2.4. Real-Time PCR Assay

The cleavage DNA from DNAzyme cleavage assay was used as the template DNA. The final 20 *μ*L PCR mixture contained 10 *μ*L of SGExcel FastSYBR Mix (Sangon Biotech), 2 *μ*L of reverse and forward primers at a final concentration of 0.5 *μ*M, 1 *μ*L of template, and 7 *μ*L of distilled water. Then, the above 20 *μ*L mixture was amplified with PCR on an ABI7500 system. The PCR amplification procedure consisted of predenaturation at 95°C for 5 min, 40 cycles of denaturation for 10 s at 95°C, and annealing for 30 s at 60°C.

### 2.5. Environmental Sample Analysis

To demonstrate the practicality of our proposed method, water samples were spiked with Pb^2+^ (1 nM, 5 nM, 10 nM, and 50 nM). These samples were tested with the same procedure used for Pb^2+^ detection. The recovery ratio was calculated based on the dPCR signals.

## 3. Results

### 3.1. Principle of the Detection System

**Figure 1:**
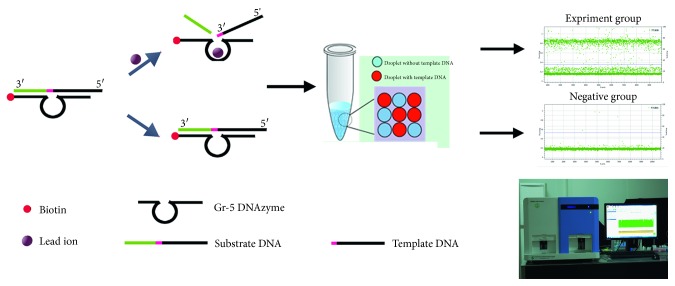
The principle of the DNAzyme biosensor detection system.

The principle of the detection system is described in Figure 1. We combined dPCR with the Gr-5 DNAzyme as the sensing system for Pb^2+^ detection. In the first step, biotin-modified Gr-5 DNAzyme was fixed on the inner wall of a streptavidin-coated plate via biotin-streptavidin interactions, the substrate strand was hybridized to the biotin-modified Gr-5 DNAzyme through complementary base pairing, and substrates that were not fixed were washed off. The substrate strand contains a single RNA base that is cleaved specifically by the classic Pb^2+^-dependent Gr-5 DNAzyme as the catalytic unit and a 50-base substrate strand extension, which is required as a template for PCR amplification. The addition of aqueous Pb^2+^ to the plate leads to cleavage of the substrate DNA at the rA site by the Gr-5 DNAzyme, resulting in the release of the cleaved substrate DNA. The cleaved substrate DNA was used as template DNA and detected with digital PCR. Consequently, the PCR amplification signal varies according to the amount of template available for amplification: the higher the concentration of aqueous Pb^2+^ was, the more the droplets with template DNA were detected. Thus, the concentration of the Pb^2+^ target can be quantified using dPCR.

### 3.2. Quantitative Assessment of Pb^2+^

**Figure 2:**
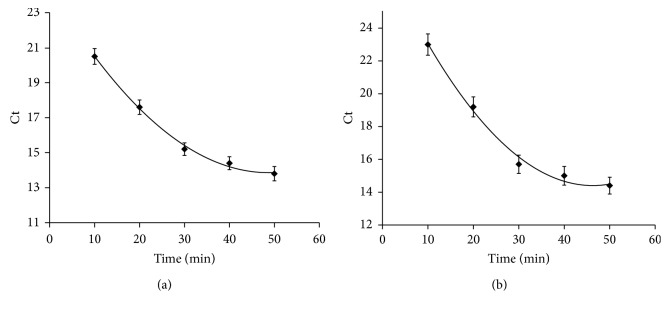
(a) Effect of the hybridization time of the detection system. (b) Effect of the substrate cleavage time of the detection system.

**Figure 3:**
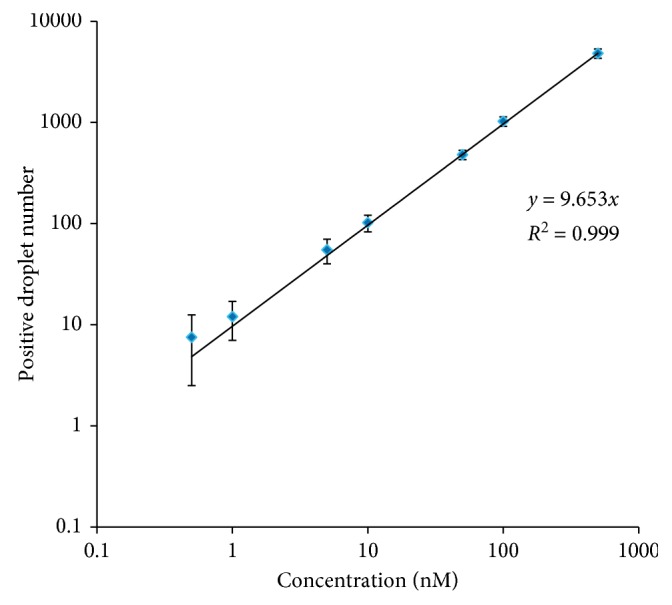
Evaluation of the detection assay by dPCR. Typical experimental result of dPCR analysis with increases in the Pb(II) concentration (0, 0.5, 1, 5, 10, 50, 100, and 500 nM).

To improve the performance of the detection system for Pb^2+^ detection, the reaction time was optimized with real-time PCR [35]. Various reaction times were investigated, and the results showed that the Ct value decreased with increasing substrate-binding time up to 30 min, indicating that sufficient binding of Gr-5 DNAzyme and substrate DNA occurred within 30 min (Figure 2(a)). In the substrate cleavage experiments in the presence of Pb^2+^, the Ct value decreased as the cleavage time increased up 30 min (Figure 2(b)). Based on these results, both the hybridization time of the Gr-5 DNAzyme and the substrate DNA and the cleavage time were set at 30 min. The concentration of DNAzyme was set at 100 nM, as described previously. Then, titration experiments were carried out to test whether the proposed method using a DNAzyme assay and dPCR can be used for Pb^2+^ quantification. There are few positive droplets in the absence of Pb^2+^, whereas there are many positive droplets when Pb^2+^ is added, suggesting that the DNAzyme was activated and catalyzed the cleavage of the substrate strand. As a result, template DNA was released, and positive droplets were generated after amplification. As shown in Figure 3, the number of positive droplets has a good linear relation with the Pb^2+^ content between 500 pM and 100 nM. This result indicated that our detection system has a dynamic range over 3 orders of magnitude and thus can achieve accurate detection of Pb^2+^ in most practical samples.

### 3.3. Specificity Assessment of the Detection System

**Figure 4:**
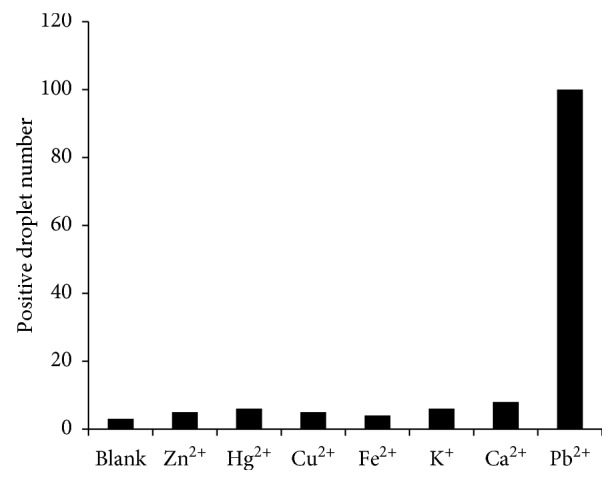
Specificity of the detection system for Pb^2+^ ions over other ions. (Pb^2+^ at 10 nM, Zn^2+^, Hg^2+^, and Cu^2+^ at 10 *μ*M, Fe^2+^ and Ca^2+^ at 1 mM, and K^+^ at 10 mM).

To evaluate the specificity of the detection system, a series of potential interference metal ions, such as Zn(II), Hg(II), Cu(II), Fe(II), K(I), and Ca(II), were tested by measuring and comparing the number of positive droplets with those in the presence of lead ions. As shown in Figure 4, only Pb^2+^ showed significantly high digital signals, while no substantial signal was produced by the remaining metal ions or the blank. These results indicate that our detection system exhibits excellent selectivity for Pb^2+^ over other environmentally relevant metal ions.

### 3.4. Detection of Pb^2+^ in Environmental Samples

We further evaluated the potential application of our detection system to real samples such as environmental substrates. Lake water samples were centrifuged to remove the insoluble materials. Then, the supernatant spiked with various concentrations of Pb^2+^ were evaluated. As shown in Table 1, the recovery for the water samples ranged from 96.2% to 105%, and the standard deviation was less than 5%. This indicated that our detection system based on the DNAzyme biosensor and dPCR offers a convenient and sensitive approach for Pb^2+^ detection in environmental substrates.

## 4. Discussion

In this study, an ultrasensitive detection method for aqueous Pb^2+^ based on digital polymerase chain reaction (dPCR) technology and a Pb^2+^-dependent DNAzyme was developed. The relationship between Pb^2+^ concentration and the number of positive droplets was established. Owing to the high sensitivity of dPCR, our proposed method exhibits high sensitivity for Pb^2+^ detection with a detection limit of 500 pM. The method also exhibits excellent selectivity due to the specificity of the Gr-5 DNAzyme. In addition, this method showed good feasibility for use in water sample detection. Previous studies have shown that many ions, such as Cu^2+^, Cd^2+^, and Na^+^, could be detected with DNAzymes [36–39]. It is very likely that this method, which combines a DNAzyme and dPCR, could be extended to other ions, which may improve its sensitivity for ion detection. In summary, the new sensor is a potential tool for Pb^2+^ detection in environmental samples.

## Figures and Tables

**Table 1:** Concentration of Pb^2+^ in environmental samples detection by the digital PCR-based detection system.

Sample	Pb^2+^ spiked (nM)	Pb^2+^ measured (nM)	Recovery (%)	RSD (%)
1	1	1.05	105.0	4.8
2	5	5.08	103.6	3.9
3	10	10.15	102.7	3.5
4	50	48.10	96.2	4.4

## Data Availability

The data used to support the findings of this study are included within the article and the supplementary informational file.

## Supplementary Materials

Supplementary Materials Supplementary Table 1: contains oligonucleotide sequences.
