# Supplementary material for: Ultrasensitive Detection of Pb2+ Based on a DNAzyme and Digital PCR
Source: J Anal Methods Chem. 2019 Jan 2;2019:3528345. doi: 10.1155/2019/3528345 (PMC6379836; doi:10.1155/2019/3528345)
Supplement: Supplementary Materials — Supplementary Table 1: contains oligonucleotide sequences. [file 3528345.f1.docx]

**Supplemental Materials**

Supplemental Table 1 Oligonucleotide sequences

| Gr-5 DNAzyme | Biotin-GGCTACGAGGGAAATGCGGTAATCATCTCTGAAGTAGCGCCGCCGTAGTG |
| --- | --- |
| Substrate DNA | AATCTGGTTTAGCTACGCCTTCCCCGTGGCGATGTTTCTTAGCGCCTTACCACTrAGGAAGAGATGATT |
| Forward Primer | AATCTGGTTTAGCTACGCCTTC |
| Reverse Primer | GTAAGGCGCTAAGAAACATCG |
